# In-Vitro and In-Vivo Tolerance and Therapeutic Investigations of Phyto-Fabricated Iron Oxide Nanoparticles against Selected Pathogens

**DOI:** 10.3390/toxics9050105

**Published:** 2021-05-08

**Authors:** Amreen Shah, Isfahan Tauseef, Manel Ben Ali, Muhammad Arfat Yameen, Amine Mezni, Amor Hedfi, Syed Kashif Haleem, Sirajul Haq

**Affiliations:** 1 Department of Microbiology, Hazara University, Mansehra 21300, Pakistan; Amreenshah36@gmail.com (A.S.); isfahan.tauseef@gmail.com (I.T.); kashifhaleem18@outlook.com (S.K.H.); 2 Department of Biology, College of Sciences, Taif University, P.O. Box 11099, Taif 21944, Saudi Arabia; mjbinali@tu.edu.sa (M.B.A.); o.zaied@tu.edu.sa (A.H.); 3 Department of Pharmacy, Abbottabad Campus, COMSATS University Islamabad, Islamabad 22060, Pakistan; arfatyameen@gmail.com; 4 Department of Chemistry, College of Science, Taif University, P.O. Box 11099, Taif 21944, Saudi Arabia; a.rachid@tu.edu.sa; 5 Department of Chemistry, University of Azad Jammu and Kashmir, Muzffarabad 13100, Pakistan

**Keywords:** therapeutic activity, iron oxide, green synthesis, histopathology, hematology

## Abstract

The *Paeonia emodi* (*P. emodi*)-mediated iron oxide nanoparticles (Fe_2_O_3_ NPs) were screened for in-vitro and in-vivo antibacterial activity against the *Staphylococcus aureus* (*S. aureus*) (ATCC #: 6538) and *Escherichia coli* (*E. coli*) (ATCC #:15224). The synthesized Fe_2_O_3_ NPs were characterized via nitrogen adsorption-desorption process, X-ray diffractometer (XRD), transmission and scanning electron microscopies (TEM and SEM), energy dispersive X-ray (EDX) and Fourier transform infrared (FTIR) spectroscopies. The S_BET_ was found to be 94.65 m^2^/g with pore size of 2.99 nm, whereas the average crystallite and particles size are 23 and 27.64 nm, respectively. The 4 μg/mL is the MIC that inhibits the growth of *E. coli,* whereas those for *S. aureus* are below the detection limit (<1.76 μg/mL). The tolerance limit of the mice model was inspected by injecting different concentration of Fe_2_O_3_ NPs and bacteria suspensions. The 14 ppm suspension was the tolerated dose and the concentration above were proved lethal. The most severe infection was induced in mice with injection of 3 × 10^7^ CFUs of both bacteria, while the inoculation of higher concentrations of bacterial suspensions resulted in the mice’s death. The histopathological and hematological studies reveals that the no/negligible infection was found in the mice exposed to the simultaneous inoculation of Fe_2_O_3_ NPs (14 ppm) and bacterial suspensions (3 × 10^7^ CFUs).

## 1. Introduction

The high frequency of bacterial infection and the developing of drug-resistant strain against the common used antibiotic is a grand change all over the world [1]. It is critical to fight against bacterial infections with conventional antibiotic therapy and thus the increasing number of resistant microbes a serious health problem [2]. Therefore, new methods and novel antimicrobial agents are needed for the treatment of incurable microbial infections to secure health around the globe [3]. The recent development in nanotechnology have enable the researchers to treat microbial infections using atomic scale particles. These extremely small particles possess high antibacterial potential to kill or reduce the bacterial colonies, provide larger surface to contact with disease causing microbes. The nanomaterials are extensively used for biomedical applications (i.e., nano-medicines, food preservation, wound dressing, protective clothing, disinfection, drug delivery and antimicrobial agents) [4]. Due to low cytotoxicity and high potency, nanoparticles are an attractive alternate of the conventional antibiotic and emerge as a new generation antibiotic [5].

The iron oxide nanoparticles are reported to have applications in targeted drug delivery, lithium ion batteries, magnetic resonance imaging (MRI), targeted destruction of tumor tissue through hyperthermia and removal of toxic metals from contaminated water [6,7,8,9]. The Fe_2_O_3_ NPs adversely affect the soil enzymatic activities of urease, catalase, phosphatase and also affect the microbial communities in a negative way. These metal oxides also exert growth inhibitory effect on the strains of Gram-negative and Gram-positive bacteria. It has also been explained in the literature that these metal oxides reduce the chlorophyll content and carotenoids along with the reduction in the length of root and shoot of rice [10]. Fe_2_O_3_ NPs can induce oxidative stress, protein aggregation and the iron accumulation and these oxide NPs can also induce neurodegeneration by inducing apoptosis [11]. Fe_2_O_3_ NPs have a moderate cytotoxic effect on lungs and can cause interstitial inflammation in lungs after pulmonary fibrosis [12]. Some of the studies have shown that the Fe_2_O_3_ NPs are converted to free iron ions after metabolism in the cells by lysosomes that affect most of the biological processes. These free ions can directly damage the mitochondria and increase the radical concentration. Another toxic effect of the Fe_2_O_3_ NPs include the significant DNA damage, along with the decreased capacity of the cells to repair DNA lesions [13]. The Fe_2_O_3_ NPs have been synthesized via sol-gel, hydrothermal, solvothermal, sonochemical, microemulsion, co-precipitation and green methods [14,15]. Among the green processes, plant mediated synthesis is a subject of great attention due to the easy handling, low cost, high yield and minimum time consumptions. Many researchers have studied in-vitro antimicrobial activity; however, very limited literature are available on in-vivo therapeutic property of Fe_2_O_3_ NPs on an animal model. Thus, a detailed study required to explore systematic toxicity and in-vivo therapeutic efficacy of Fe_2_O_3_ NPs against disease causing pathogens.

In current study, *P. emodi* leaves extract was used as reducing and capping agent for the green synthesis of Fe_2_O_3_ NPs. The *P. emodi* belongs to the family Paeoniaceae found in the hilly area of northern area of Pakistan and locally used for the treatment of colic, asthma and hypertension. The physiochemical properties of the synthesized Fe_2_O_3_ NPs were investigated through N_2_ adsorption-desorption, XRD, SEM, TEM, FTIR and EDX techniques. The MIC and in-vitro experiment was performed using the 96-well plate and agar well diffusion methods respectively. The in-vivo tolerance of the mice model was examined by inoculation of different concentrations of the selected bacteria and Fe_2_O_3_ NPs. The synergistic effect of bacteria and Fe_2_O_3_ NPs on the mice model was also observed by execution of histopathological and hematological analysis.

## 2. Materials and Methods

### 2.1. Chemical Used

The analytical grade chemicals, including iron sulphate heptahydrates, iodonitrotetrazolium chloride ethanol and agar nutrients, were purchased from Sigma-Aldrich. All the working solutions were prepared in deionized water whereas the Fe_2_O_3_ NPs suspensions for in-vitro and in-vivo were prepared in normal saline. The glassware were washed with 15 % (*v*:*v*) nitric acid solution and rinsed with deionized water. The selected bacterial pathogens were obtained from the department of Pharmacy.

### 2.2. Preparation of P. emodi Leaves Extract

The collected healthy *P. emodi* leaves were washed with ordinary water, followed by deionized water, in order to remove dust particles. The leaves were shade dried to avoid any damage to chlorophyll and other chemical constituents. The leaves were then chopped into fine pieces with a sterile chopper and 10 g of the chopped leaves was added into a titration flask containing 200 mL of boiled deionized water. The titration flask was tightly covered with aluminum foil for 12 h. The light-green crude mixture was filtered and centrifuged (at 4000 for 15 min) and the clear upper layer was stored at 4 °C.

### 2.3. Synthesis of Fe_2_O_3_ NPs

For the synthesis of Fe_2_O_3_ NPs, a stock solution (5 mM) was prepared by dissolving 0.72 g in 0.5 L of deionized water. For each reaction, 15 mL of the extract was slowly added into 45 mL of precursor solution with constant stirring at 50 °C. After 60 min, the reaction mixture was aged for 24 h at room temperature and the suspended particles were collected via centrifugation. The solid product obtained was washed with deionized water followed by ethanol and were dried at 100 °C and stored in an airtight polyethylene bottle.

### 2.4. Characterization

The GeminiVII2390i instrument was run to perform the N_2_ adsorption-desorption experiment and the data obtained were manipulated by BET and BJH methods to deduced texture parameters (surface area and pore size). The Panalytical X-pert Pro was operated in the 2θ range of 20°–80° to determine the crystallographic parameters, whereas the full width and half maxima (FHWM) values were considered to calculate crystallite size. The surface morphology was studied via SEM model 5910, JEOL, Tokyo, Japan and TEM (HRTEM, JEM-2010) provided by Field Electron and Ion Company (FEI), Cleveland, TN, USA. The EDX coupled with SEM (model: INCA 200, Oxford Instruments, Oxfordshire, UK) was used to analyze the elemental composition, whereas the surface chemical moieties were identified via FTIR analysis (Nicolet 6700, Thermo Fisher Scientific, Waltham, MA, USA).

### 2.5. Minimum Inhibitory Concentration Assay

The minimum inhibitory concentration (MIC) experiment was carried via the 96-well plate method, also called ELISA, and 100 μL of broth media was transferred to 72 wells of column 1 to 9. The 100 μL of Fe_2_O_3_ NPs (1024 μg/mL) in triplicate in 48 wells (column 1 to 6), whereas the antibiotic solution (256 μL/mL) was poured into 24 wells (column 10 to 12) in triplicate. The dilution was done by taking 100 μL from the first row and added into the second row and so on, whereas the 100 μL taken from the last row was discarded. The microbial suspension *E. coli* and *S. aureus* in normal saline and compared with 0.5 MacFarland standard and the 100 μL bacterial suspension. The *S. aureus* suspension was added in the first three columns, whereas the next column was loaded with *E. coli* suspension. The 40 μL iodonitrotetrazolium chloride (INT) solution (1 mg/5 mL) was added into the columns containing bacteria suspension, which act as indicator for the non-inhibitory concentration of Fe_2_O_3_ suspension. Then, after the addition of 40 μL of INT solution and 100 μL bacterial suspension, the final concentration of the stock suspension was reduced to 898.25 μL/mL from 1024 μg/mL. The MIC value was counted at the lowest concentration in the row just before the row where the color change occurred. The ciprofloxacin was used as the positive control to compare the activity/efficacy of Fe_2_O_3_ NPs.

### 2.6. In-Vitro Antibacterial Assay

The agar well diffusion method was used to examine the in-vitro antibacterial efficacy of Fe_2_O_3_ NPs against the *E. coli* (ATCC #:15224) and *S. aureus* (ATCC #: 6538), where agar nutrient was used as medium for antibacterial susceptibility test. The 0.5 McFarland solution was used for clinical strain where Fe_2_O_3_ stock suspension was prepared by dispersing 1, 2, 3 and 4 mg in 1 mL in normal saline. The wells with diameter of 8 mm were bored with sterile borer and bacterial pathogen was streaked on media with help of sterile swabs. Afterward, 100 μL of each suspension was added into each well independently and were incubated at 37 °C. The zone of inhibition was measured in millimeter (mm) after 24 h was consider is the activity of Fe_2_O_3_ NPs against selected bacteria.

### 2.7. Tolerance Study

All animal experiments were performed in compliance with the local ethics committee. The female mice that were aged between 6 to 8 weeks and weighed 20–22 g were randomly divided into five groups (*n* = 5 for each group). Effect of Fe_2_O_3_ NPs concentration on the mice model was examined by injecting 0.066 mL of 5, 10, 15, 20 and 25 ppm suspension into five mice groups (*n*= 5 for each group) and their behavior observed for 14 days. Similarly, to test the tolerance of mice against bacterial pathogens, five groups of mice (*n* = 5) were individually inoculated with different suspensions (1 × 10^7^, 2 × 10^7^, 3 × 10^7^, 4 × 10^7^ and 5 × 10^7^ CFUs) of both pathogens and examined for fourteen days. Initially, the behavior of the mice model was examined for four hours, after which it was checked every 8 h for 14 days, to see any unusual behaviors, eating, drinking and weight. The maximum dose of nanoparticles and the bacterium suspension on which mice survive for 14 days were utilized for further studies.

### 2.8. In-Vivo Antibacterial Study

The in-vivo therapeutic activity of Fe_2_O_3_ NPs against *S. aureus* and *E. coli* was evaluated upon the simultaneous inoculation of 14 ppm and 3 × 10^7^ CFUs suspensions of Fe_2_O_3_ NPs and bacterial pathogens. The histopathological examination of kidney, spleen, lung and liver was performed after the simultaneous inoculation of Fe_2_O_3_ NPs and pathogen suspension. The healthy and pathogen-free mice that had an age between 6 to 8 weeks and weighed 20–22 g were selected for in-vivo antibacterial studies. A 0.066 mL of Fe_2_O_3_ NPs suspension (14 ppm) and bacterial suspensions (3 × 10^7^ CFUs) were administered simultaneously into the mice model. The efficacy of Fe_2_O_3_ NPs were examined by comparing the histopathological and hematological results of infected mice with those inoculated with both bacteria and Fe_2_O_3_ NPs at the same time.

### 2.9. Histopathological Study

The kidney, spleen, liver and lung were chosen for the histopathological study and a small portion of these organs were treated with 10% formalin followed by the paraffin, and cut down into 5–6 mm (mm) slices and fixed on the glass microscopic slide utilizing the standard histopathological process [12]. The hematoxylin eosin was used to stain the sections and examined through light microscope.

### 2.10. Hematological Assay

The blood sample taken from the heart of mice infected with bacterial species and those injected with the specific amount of both Fe_2_O_3_ NPs and bacteria suspensions were measured after anticoagulation. The automatic hematology analyzer (MEK-6318K) was used to study the blood parameters like red blood cell (RBC), red blood cell volume distribution width (RDW), white blood cell (WBC), hemoglobin (HGB), mean corpuscular hemoglobin (MCH), mean corpuscular hemoglobin concentration (MCHC), mean corpuscular volume (MCV), platelet count (PLT), mean platelet volume (MPV), mononuclear percentage (MOD%), lymphocyte percentage (LYM%) and granulocyte percentage (GRN%).

## 3. Results

### 3.1. Physicochemical Study

#### 3.1.1. Surface Area and XRD Analysis

**Figure 1:**
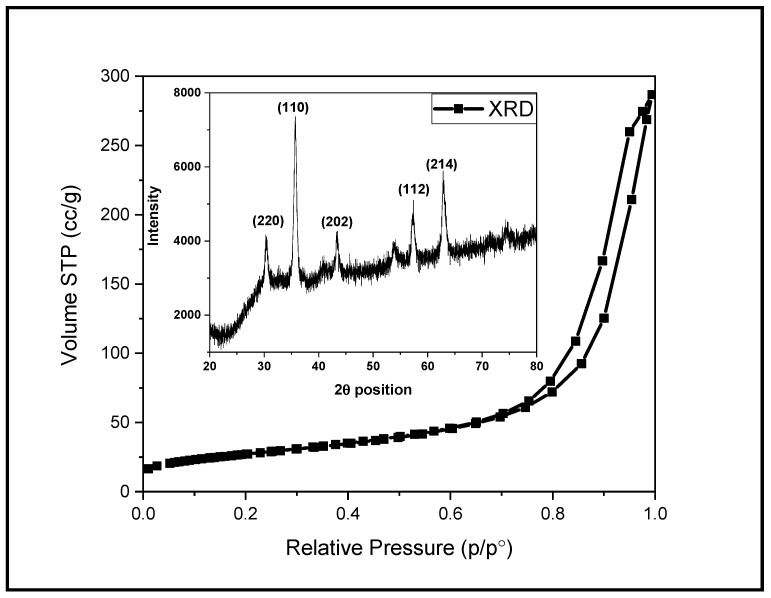
N_2_ adsorption-desorption isotherm (inset: XRD pattern) of Fe_2_O_3_ NPs.

The reversible type II N2 adsorption/desorption isotherm obtained for Fe_2_O_3_ NPs shows maximum adsorption in the range of 0.75 to 9.8 p/po, forming the H3 hysteresis loop. Usually this type of isotherm is obtained for the aggregates of plate-like particles. The surface area calculated by BET method is 94. 65 m^2^/g, whereas the BJH pore size along with pore volume are found to be 2.99 nm and 0.443 cc/g, respectively. The XRD technique was employed to find out the structural properties of Fe_2_O_3_ NPs (inset: Figure 1). The X-ray diffraction pattern confirmed the crystalline nature of Fe_2_O_3_ NPs as sharp diffraction peaks were observed. The diffraction peaks for Fe_2_O_3_ NPs at 2θ position are 30.34°, 35.71°, 43.32°, 53.98°, 62.88° and 74.29° and correspond to the hkl values of (220), (110), (202), (112) and (214), respectively. All these diffraction peaks are matching with the standard reference card 01-071-6336 and are associated with Fe_2_O_3_ NPs with face-centered cubic geometry. The average crystallite size estimated by Scherer’s formula is 23 nm.

#### 3.1.2. SEM and TEM Analysis

**Figure 2:**
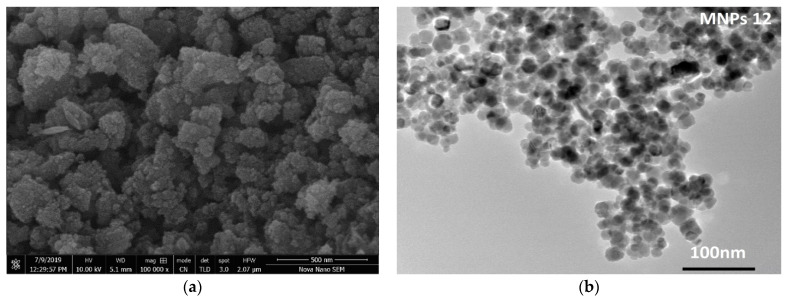
SEM (**a**) and TEM (**b**) micrographs shows the surface morphology of Fe_2_O_3_ NPs.

The SEM and TEM techniques are operated to analyze the surface features of Fe_2_O_3_ NPs as shown in Figure 2a,b. The SEM image Figure 2a reveals that the agglomeration of small particles led to the formation of bunch like structure. However, due to the aggregation of small particles, it is difficult to identify the specific shape and size of particles from SEM micrograph. The TEM micrograph (Figure 2b) explains the physical appearance of Fe_2_O_3_ NPs, where the majority of the particles exhibit a spherical/nearly spherical shape with an almost smooth surface. The mono-disperse particles are interconnected with each other led to the formation of a larger network of small particles. The particles with clear boundaries are considered to estimate the particles size and are found in the range of 22.05 to 34.26 nm with an average size of 27.64 nm.

#### 3.1.3. FTIR and EDX Analysis

**Figure 3:**
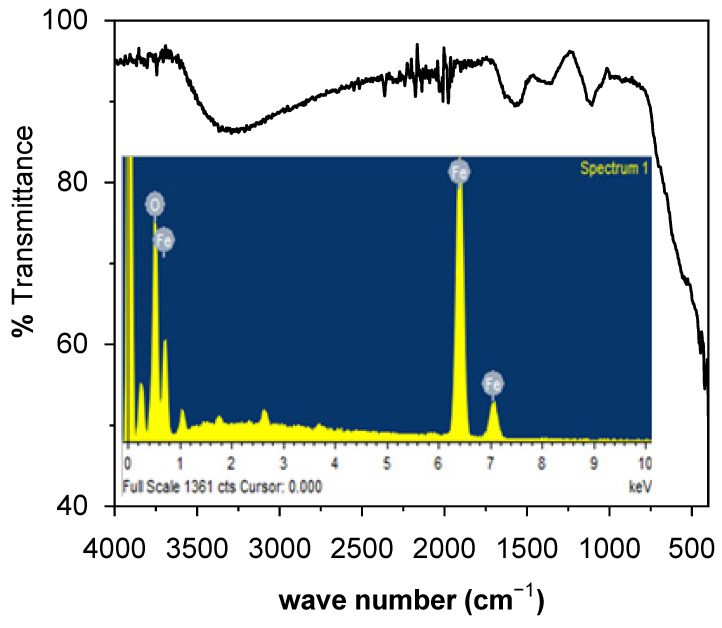
FTIR spectrum (inset: EDX spectrum) of Fe_2_O_3_ NPs.

The FTIR spectrum of Fe_2_O_3_ NPs, shown in Figure 3, possess a broad absorption band at 3000–3500 cm^−1^ and a weak band at 1200 cm^−1^, which are associated to the stretching vibration of hydroxyl groups [13]. A weak vibration at 570 cm^−1^ as well as a small band at 800 cm^−1^ can be assigned to the bending vibration of iron and oxygen in Fe_2_O_3_ NPs [10]. The EDX of Fe_2_O_3_ NPs (inset: Figure 3) exhibit sharp characteristic peaks at 0.8, 6.4 and 7.1 keV, attributed to the presence of iron along an intense peak at 0.5 keV due to oxygen. The data reveals that Fe_2_O_3_ NPs contain a 61.68% weight of iron with a 38.32% weight of oxygen. The purity of Fe_2_O_3_ NPs can; therefore, be claimed as only the peaks associated to iron and oxygen were detected.

### 3.2. In-Vitro Therapeutic Study

#### 3.2.1. Minimum Inhibitory Concentration

**Figure 4:**
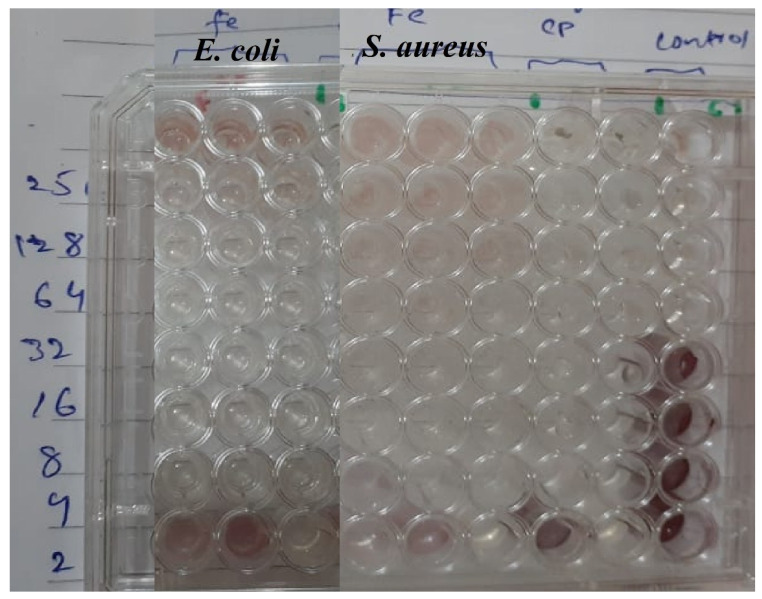
Minimum inhibitory concentration of Fe_2_O_3_ NPs against *S. aureus* and *E. coli.*

The quantitative estimate of Fe_2_O_3_ NPs for antibacterial activity was evaluated by performing minimum inhibitory concentration (MIC) analysis against the selected pathogen as shown in Figure 4. The colorless wells shows that the amount of Fe_2_O_3_ NPs was capable to inhibit bacterial growth where color wells indicate the inhibition was unsuccessful. The concentration in the colorless well just before the colored wells is count as MIC against that organism. The color change in the well containing 1.76 μg/mL shows that the 3.51 μg/mL of Fe_2_O_3_ NPs was the minimum amount that inhibit the growth of *E. coli*. The last wells were not turn colored suggest that the MIC of Fe_2_O_3_ NPs against *S. aureus* is the detection limit (<1.76 μg/mL). The results shows the concentration dependent activity against selected pathogens and suggest that Fe_2_O_3_ NPs have strong inhibiting power against *S. aureus* as compared *E. coli*.

#### 3.2.2. In-Vitro Antibacterial Activity

**Figure 5:**
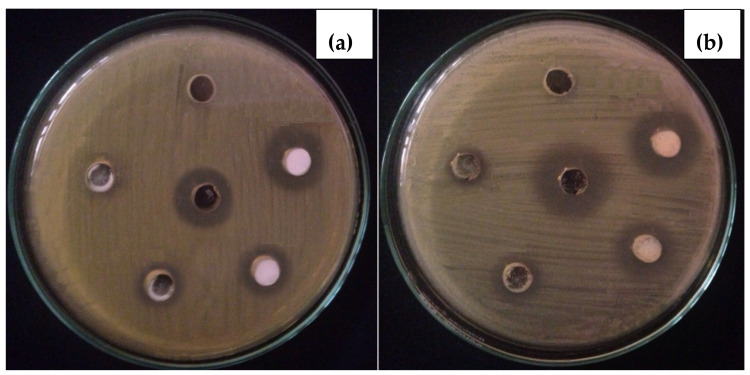
Experimental photographs of in-vitro activity of Fe_2_O_3_ NPs against *E. coli* (**a**) and *S. aureus* (**b**).

The Fe_2_O_3_ NPs was screened against *S. aureus* and *E. coli* following agar well diffusion process and the clear inhibition zones was measured is the activity synthesized materials. The experimental photographs given in Figure 5, shows that with increasing concentration of Fe_2_O_3_ NPs suspension, the growth inhibiting capability also increases. Previously the dose dependent antibacterial activity of Fe_2_O_3_ NPs was conducted, where the zone of inhibition for *S. aureus* was 7 mm at 5 mg/mL, but it nearly doubled to 12.5 mm at 30 mg/mL. *Klebsiella* spp., on the other hand, is tolerable at doses between 5 and 20 mg/mL, although 30 mg/mL doses result in an inhibition zone of around 9 mm [16]. Base on the inhibition zones, it was seen that *S. aureus* shows less resistance to Fe_2_O_3_ NPs as compared to *E. coli*. This was also proven in the MIC experiment that the growth of *S. aureus* bacteria was inhibits lower Fe_2_O_3_ NPs concentration (<1.76 μg/mL), whereas 3.51 μg/mL was required to stop growth of *E. coli*. Cell wall composition, surface charge, rigidity and permeability are the key factors that affect the activity of any antibacterial agent [3]. The phosphate group of teichoic acid exist in cell wall of Gram-positive bacteria, whereas phospholipids and lipopolysaccharide present in the cell wall of Gram-negative bacteria are responsible for surface negative charge [2].

Several reactive species present in the aqueous suspension of Fe_2_O_3_ NPs, including metal cation, hydroxyl radical and superoxide radical anion contributing to the antibacterial action of MO NPs. The Fe_2_O_3_ NPs have the ability to produce the iron cation (Fe^3+^) in solution, which accumulated on the surface of bacteria due to the electrostatic forces of attraction. The Fe^3+^ cation penetrates inside to disturb the cytoplasmic composition and inhibit the growth of microorganisms [4]. The antimicrobial action of Fe_2_O_3_ NPs is also because of hydroxyl radical and superoxide present in the solution. The dissolved oxygen is a major source for the production of superoxide radical, which reacts with polyunsaturated compounds of the outer membrane, causing a significant damage to the cell by initiating self-propagation chain reactions. The hydroxyl radical is an aggressive oxidant and denature several biological molecules present in the microorganisms [14]. The reaction of water and hydroxyl radical led to the production of hydrogen peroxide, which penetrate inside the bacterium cell and disturb the cytoplasmic composition and prove lethal for microbial life [15].

### 3.3. Tolerance and Therapeutic Study

#### 3.3.1. Histopathological Analysis

**Figure 6:**
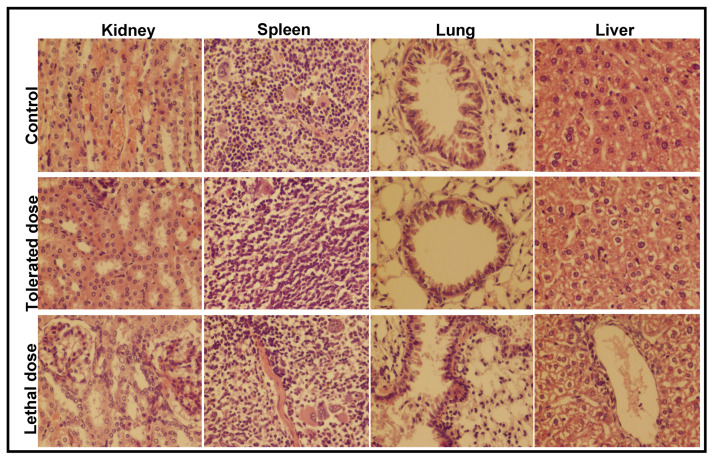
The histopathological examination of the kidney, spleen, lung and liver of mice after injecting a low and a high dose of Fe_2_O_3_ NPs for 14 days; tolerated dose (14 ppm) and lethal dose (20 ppm).

Effect of Fe_2_O_3_ NPs concentration on mice model was examined by injecting 5, 10, 15, 20 and 25 ppm into five mice groups (*n* = 5 for each group) and observed their behavior for 14 days. The mice inoculated with 20 and 25 ppm are either expired on the spot or with passage of time, whereas those exposed to 10 ppm live for 14 days. Extreme skin infection was examined in mice feed with 15 ppm suspension. The mice were seemingly disturbed and gradual weight loss was observed, which may be due to the decreased appetite. The strange behavior of mice continued till day 14, whereas two members of this group died after 11 days. This shows that the concentration above 15 ppm was not significant under the current reaction condition. Afterward, four groups of mice were inoculated with 11, 12, 13, 14 ppm to find the exact tolerated dose of Fe_2_O_3_ NPs. The mice in the groups injected with 11 and 12 ppm were normal till day 14, took normal food and performed usual activities. The mice dosed with 14 ppm suspension lived till day 14; however, skin infection increased and minor weight loss was observed up to day nine. Later on, the mice used took normal food and lead a normal life with no unusual behavior. The control group, tolerated group (14 ppm; where the infected mice become normal after few days) and the high dose group (20 ppm; lethal dose led to death) were subjected to histopathological examination to evaluate the effect of Fe_2_O_3_ NPs on the different organs of mice. The results, shown in Figure 6, reveal the comparative histopathological observation of the kidney, spleen, lung and liver of the mice.

The kidney section, shown in Figure 6, aside from a mild congestion, showed no major difference between the control group and those exposed to the tolerated dose. The kidney of mice inoculated with the lethal dose shows lesions with a different degree of hemorrhages and necrotic foci due to the ischemic and nephrotoxic agent. The degenerative changes in tubular epithelial cells were observed with frequent leukocytic infiltrations. The metabolic irregularity in mice was due to the necrosis in the tubular epithelium [16,17,18]. The histopathological study shows that the spleen seemed normal after 14 days post injection of the tolerated dose, and a minor hemorrhage was observed in the parenchyma of spleen. The extensive leukocyte infiltration was seen in the splenic parenchyma of mice inoculated with the lethal dose, whereas crippled blood vessels were seen in both spleen sections. The slight inflammation with broadened alveolar septum and a rounded shaped bronchiole was observed in the lung of mice injected with 14 ppm suspension of Fe_2_O_3_ NPs. The coagulation in the epithelial linings with complete damage of bronchi, leading to extensive leukocytic infiltration, was observed in the lung section of mice inoculated with 20 ppm Fe_2_O_3_ suspension [18,19]. The few alveoli filled with fibrin were ruptured and coalesced together to form a bullae [20]. Chronic inflammation in the alveolar septum was seen, leading to pathological lesion and, ultimately, resulting in complete damage [21]. Beside the minor congestion and hemorrhages, the hepatocytes are seen to be normal in the liver section at low dose. Extensive lesions with moderate hemorrhaging were found in the liver after dosing with 20 ppm suspension of Fe_2_O_3_ NPs [18,22].

**Figure 7:**
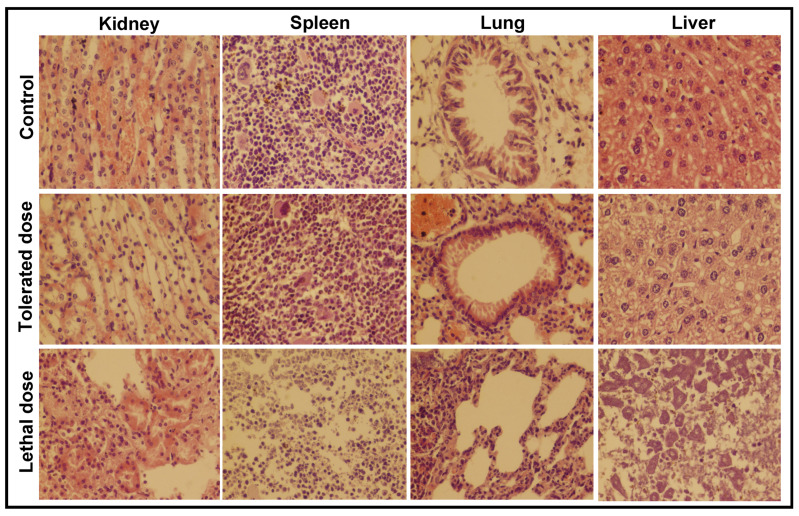
The histopathological examination of the kidney, spleen, lung and liver of mice after injecting a low and a high dose of *S. aureus* inoculums for 14 days; (tolerated dose = 3 × 10^7^ CFUs; lethal dose = 4 × 10^7^ CFUs).

**Figure 8:**
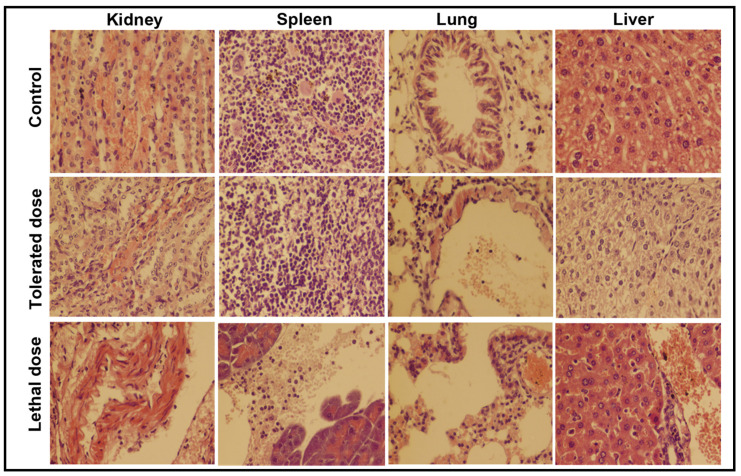
The histopathological examination of the kidney, spleen, lung and liver of mice after injecting a low and a high dose of *E. coli* inoculums for 14 days; (tolerated dose = 3 × 10^7^ CFUs; lethal dose = 4 × 10^7^ CFUs).

To observe the tolerance of mice against bacterial pathogens, five groups of mice (*n* = 5) were individually inoculated with different suspensions (1 × 10^7^, 2 × 10^7^, 3 × 10^7^, 4 × 10^7^ and 5 × 10^7^ CFUs) of *S. aureus* and examined for fourteen days. The histopathological examination of the kidney, spleen, lung and liver was carried out for two groups—the group injected with 3 × 10^7^ CFUs (tolerated dose) and the group injected with 4 × 10^7^ CFUs (lethal dose)—and the microscopic results are shown in Figure 7. The same experiment was repeated to examine the tolerance of mice against *E. coli* and the microscopic images are given in Figure 8. The mice loaded with lethal dose died with the passage of time and showed sudden weight losses, unusual behavior and extreme skin infections. The mice administered with 3 × 10^7^ CFUs showed minor skin infection, dehydration and weight loss, a low intake of food and uncontrolled breathing with unusual behavior.

The kidney of mice injected with a tolerated dose of both bacterial suspensions showed mild congestion and necrosis on the parenchymal surface, whereas degeneration in the glomeruli and extensive necrosis with a high degree of infiltrations were seen in mice exposed to the lethal dose. The spleen of mice exposed to *S. aureus* (3 × 10^7^ CFUs) was almost normal, whereas mild hemorrhaging was found in the spleen of mice injected with 3 × 10^7^ CFUs of *E. coli*. Extreme leukocytic infiltration in the parenchyma and hemorrhaging on the capsular spleen surface were seen after the inoculation of 4 × 10^7^ CFUs of both bacteria. In lung cells, a slightly broadened alveolar septum and lymphocyte infiltration were seen at low dose, whereas, at high dose, extensive widening and huge lymphocyte penetration resulted in the complete damage of lung cells [23]. The tissue of the studied organs of the mice administered with the tolerated dose were found to be normal/nearly normal in comparison with the control group. The histopathological observation discloses the extreme abnormal condition of mice organs exposed to the lethal/high concentration of Fe_2_O_3_ NPs and bacterial inoculums.

**Figure 9:**
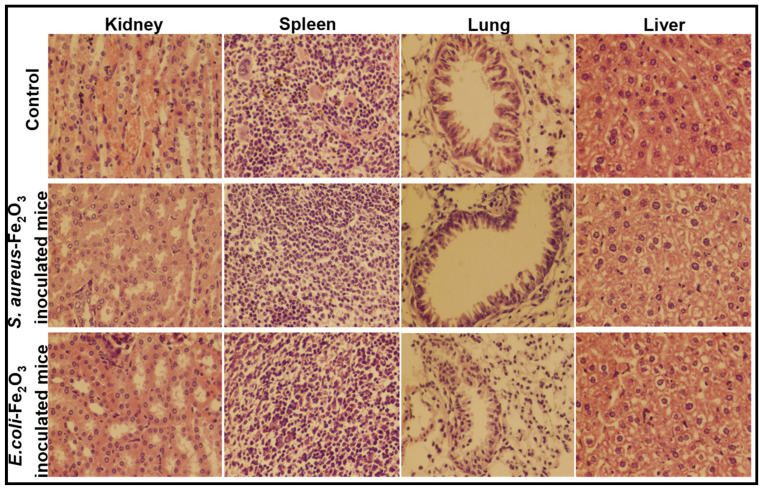
The histopathological examination of the kidney, spleen, lung and liver of mice after the simultaneous inoculation of the tolerated concentration of both Fe_2_O_3_ NPs (14 ppm) and bacterial suspension (3 × 10^−7^ CFUs).

The in-vivo therapeutic activity of Fe_2_O_3_ NPs against *S. aureus* and *E. coli* was evaluated upon the simultaneous inoculation of 14 ppm and 3 × 10^7^ CFUs suspensions of Fe_2_O_3_ NPs and bacterial pathogens (Figure 9). The histopathological examination of the kidney, spleen, lung and liver was performed after the simultaneous inoculation of Fe_2_O_3_ NPs (14 ppm) and bacterial pathogens (3 × 10^7^ CFUs) as shown in Figure 8. After the vaccination of Fe_2_O_3_ NPs and bacterial suspension at the same time, the mice were seemingly disturbed and had no attraction for food for a few days. The mice became normal with the passage time and took normal food. The therapeutic results reveal that the severe infections that were experienced during the tolerance study of mice against bacteria were not observed. However mild changes were experienced during the histopathological observations. An insignificant congestion and hemorrhaging with no morphological symptoms were seen in the kidney and liver of mice exposed to *S. aureus* and Fe_2_O_3_ NPs at the same time, respectively. The histopathology also suggests that minor changes were found in the mice exposed to *E. coli* and Fe_2_O_3_ NPs. A mild congestion in the kidney along with insignificant hemorrhaging in the liver were observed. A slightly suppressed respiratory bronchiole with alveoli appeared in the lung section, whereas the liver section was almost normal.

#### 3.3.2. Hematological Analysis

The hematological study was conducted for the mice infected with bacteria and those inoculated with bacteria and Fe_2_O_3_ NPs at the same time. The blood samples taken from the heart of mice were measured after anticoagulation. The blood parameters, like WBC, RBC, HGB, MCV, MCH, MCHC, RDW, PLT, MPV, LYM%, MOD%, GRN%, were determined by an automatic hematology analyzer (MEK-6318K). The blood hematological study reveals several significant changes for the mice exposed to both Fe_2_O_3_ NPs and pathogenic suspension. Compared with normal values, some blood parameters, including WBC, MON#, GRAN#, Lymph, MON%, GRAN%, RDW and PLT, of the infected mice were significantly high, whereas a valuable decrease was seen in MCV, MCH and MCHC as compared to the normal values, as shown in Table 1. The nanoparticles are believed to enter into the lungs and, by breaking the alveolar cell, were transferred into the blood circulating system and disturbed the hematological system [19,24].

The high count of WBC (leukocytosis) is a sign of emotional and physical stress and shows that the immune system is working against an infection. It is also reported that the organism with particular blood cancer may also have a high WBS count. The idiopathic hyper eosinophilic syndrome can cause damage to the heart, liver, lungs, skin and nervous system and the organism may experience weight loss, swelling, skin rash, confusion, weakness and coma [25]. The severe infections are responsible for monocytosis, which is a common sign of chronic myelomonocytic leukemia, where the blood is produced in the bone marrow. However some recent investigations reveal that the higher monocyte number is related to some cardiovascular diseases [18,26]. Granulocytes are WBCs that possess small granules which help to fight against viral and bacterial infection. The number of granulocytes (granulocytosis) increases for infections, autoimmune diseases and blood cell cancer and the organism experiences abnormal breeding, pale skin, excessive sweating during sleeping, fatigue and loss of appetite [27].

The amount of red blood cells (RBCs) can be measured via the red blood distribution width (RDW) blood test, and the RDW score above the normal range is due to nutrient deficiency (iron, vitamin B-12 and folate) and infections, and can also be linked to anemia, cancer, heart and liver diseases [19]. High RDW with low MCV indicates microcytic anemia related to the deficiency of RBCs and will be smaller than the normal value [21]. The Alanine aminotransferase (ALT) is released into the blood stream due to the damage of the liver cell and the organism may experience jaundice, loss of appetite, tiredness, abdominal pain, nausea and vomiting. The high ALT indicates liver damage due to hepatitis, liver cancer, cirrhosis and the intake of medicines that cause liver infections [28]. The hematocrit test (HCT) is carried out to estimate the proportion of RBCs in the body, and higher values are because of dehydration, polycythemia and lung/heart disorders. The mean MCV, which is the average RBCs’ size, and the range of MCV shows the type of anemia that the organism experienced. The microcytic anemia is the lower MCV, where the RBCs’ sizes are too small, which are due to lead poisoning, chronic disease, thalassemia and iron deficiency (poor dietary intake of iron, menstrual/ gastrointestinal bleeding) [29]. The mean MCH is the average quantity of hemoglobin in RBCs in the body. The microcytic anemia, celiac disease and iron deficiency are the main causes of the low MCH and symptoms like shortness of breath, loss of regular stamina, dizziness, regular tiredness, weakness in the body and some skin infections may also appeared, when the MCH levels fall too low [30]. The mean MCHC is the normal concentration of hemoglobin inside a single RBC. The organism with low MCHC levels may have fatigue, shortness of breath, pale skin, easily bruised, dizziness and weakness. The most common cause of low MCHC is hypochromic microcytic anemia, which is due to the lack of iron, hemolysis and peptic ulcer. The organism with this type anemia experience rapid breathing, fast heart rate, sweating, confusion, coughing, wheezing and shortness of breath [31]. All these observations suggest that the infection was caused in mice due to insertion of bacterial pathogens. Thus, an attempt was made to examine the in-vivo therapeutic effect of Fe_2_O_3_ NPs against bacterial infections, and the data obtained from the hematological analysis of the blood samples from the extremely infected mice (no death) treated with Fe_2_O_3_ NPs. The results show that the increased/decreased values of WBC, MON#, Lymph, MON% and MCV were found almost in the normal range, which confirm the therapeutic significance of Fe_2_O_3_ NPs. The deviated values of GRAN%, PLT, RDW, MCH and MCHC tended toward the normal range after the simultaneous inoculation of Fe_2_O_3_ NPs and bacterial pathogens. All these results prove the in-vivo therapeutic efficacy of Fe_2_O_3_ NPs against *S. aureus* and *E. coli* in a mouse model.

## 4. Conclusions

An easy and eco-friendly attempt was made for the synthesis of Fe_2_O_3_ NPs and the highly crystalline nature and nano-range particles was confirmed through different physicochemical techniques. The MIC concentration for *S. aureus* was found to be less than 1.76 mg/L, whereas that for *E. coli* was 3.51 mg/L. The in-vitro study shows that *E. coli* shows greater resistance against the Fe_2_O_3_ NPs, as compared to *S. aureus*. The in-vivo tolerance study shows that the mice sustain up to 14 ppm suspension of Fe_2_O_3_ NPs and 3 × 10^7^ CUFs of bacteria, whereas the higher concentration induced extreme histological lesion, congestion, hemorrhaging and complete damage of bronchi, which, ultimately, led to the death of the mice. The deviated values of some blood parameters confirm the infection was caused due to the inoculation of bacteria. However, the histopathological and hematological investigations reveals that no/negligible infection was found in the mice after simultaneous injection of Fe_2_O_3_ NPs and bacterial suspension. Thus, this study explores the therapeutic significance of Fe_2_O_3_ NPs and reveals that, up to a specific concentration, Fe_2_O_3_ NPs could be an excellent candidate for both in-vitro and in-vivo antibacterial activity.

## Figures and Tables

**Table 1:** Hematological analysis infected and treated mice models.

Parameters	*E. coli* Infected Mice	*E. coli*-Fe_2_O_3_ Inoculated Mice	*S. aureus* Infected Mice	*S. aureus*-Fe_2_O_3_ Inoculated Mice	Normal Range
WBC	13.7 × 10^9^/L	1.7 × 10^9^/L	8.8 × 10^9^/L	2.9 × 10^9^/L	0.8–6.8
Lymph #	4.2 × 10^9^/L	1.2 × 10^9^/L	4.7 × 10^9^/L	2.3 × 10^9^/L	0.7–5.7
Mon #	1.2 × 10^9^/L	0.1 × 10^9^/L	0.6 × 10^9^/L	0.1 × 10^9^/L	0.0–0.3
Gran #	8.4 × 10^9^/L	0.4 × 10^9^/L	3.5 × 10^9^/L	0.5 × 10^9^/L	0.1–1.8
Lymph %	30.0%	71.5%	53.0%	79.8%	55.8–90.6
Mon %	8.8%	3.0%	7.7%	3.2%	1.8–6.0
Gran %	61.1%	25.5%	39.7%	17.0%	6.36–9.42
RBC	9.24 × 10^12^/L	7.02 × 10^12^/L	8.45 × 10^12^/L	8.61 × 10^12^/L	11.0–14.3
HGB	11.1 g/dL	10.9 g/dL	13.2 g/dL	11.8 g/dL	8.6–38.9
HCT	39.6%	37.3%	44.9%	42.0%	34.6–44.0
MCV	42.9 fL (L)	53.2 fL (L)	53.0 fL	48.8 fL (L)	48.2–58.3
MCH	12.0 pg (L)	15.5 pg (L)	15.5 pg (L)	13.7 pg (L)	15.8–19.0
MCHC	28.0 g/dL (L)	29.2 g/dL (L)	29.3 g/dL (L)	28.0 g/dL (L)	30.2–35.3
RDW	20.3% (H)	17.5% (H)	21.1% (H)	16.3% (H)	13.0–17.0
PLT	2599 × 10^9^/L (H)	1119 × 10^9^/L (H)	1016 × 10^9^/L (H)	143 × 10^9^/L (H)	450–1690
MPV	4.9 fL	6.0 fL	5.5 fL	6.1 fL	3.8–6.0
PDW	15.7	16.7	16.6	18.2	--
PCT	--	0.671	0.558%	0.087	--

White blood cell (WBC), red blood cell (RBC), red blood cell volume distribution width (RDW), hemoglobin (HGB), mean corpuscular hemoglobin (MCH), mean corpuscular hemoglobin concentration (MCHC), mean corpuscular volume (MCV), platelet count (PLT), mean platelet volume (MPV), mononuclear percentage (MOD%), lymphocyte percentage (LYM%) and granulocyte percentage (GRN%). % = percentage, # = counts.
